# The Effects of Spiritual Wellbeing on Self-Perceived Health Changes Among Members of the Church of England During the COVID-19 Pandemic in England

**DOI:** 10.1007/s10943-023-01790-y

**Published:** 2023-04-13

**Authors:** Andrew Village, Leslie J. Francis

**Affiliations:** 1 School of Humanities, York St John University, York, UK; 2 Centre for Educational Development, Appraisal and Research (CEDAR), University of Warwick, Coventry, CV4 7AL UK; 3 World Religions and Education Research Unit (WRERU), Bishop Grosseteste University, Lincoln, UK

**Keywords:** Affect, COVID-19, England, Health, Spiritual wellbeing, Psychological wellbeing

## Abstract

This paper tests whether changes in spiritual wellbeing were correlated with self-rated changes in mental and physical health after controlling for changes in psychological wellbeing in a sample from the Church of England taken during the third national COVID-19 lockdown in 2021. During the third lockdown in England an online survey, named *Covid-19 and Church-21*, was delivered through the Qualtrics XM platform from 22 January to 23 July 2021. The responses included 1878 Anglicans living in England. The change in spiritual wellbeing scale was produced using self-reported changes in the frequency of key spiritual practices (prayer and Bible reading), trust in God, the quality of spiritual life, and spiritual health. Changes in mental and physical health were assessed using single self-report items. Changes in psychological wellbeing were assessed using the Index of Balanced Affect Change (TIBACh). After controlling for changes in psychological wellbeing, better change in spiritual wellbeing was positively correlated with better change in both mental and physical health. Negative affect may have mediated the relationship between spiritual wellbeing and both mental and physical health, and positive affect may also have mediated the relationship with mental health. The results suggest changes in spiritual wellbeing, as defined within a Christian religious context, may have had positive effects in promoting better mental and physical health during a sudden crisis such as the COVID-19 pandemic.

## Introduction

The COVID-19 pandemic severely disrupted the religious life of millions of people around the world (Cavaliere, 2021; Edelman et al., 2021; Hashim et al., 2021; Johnston et al., 2022; Osei-Tutu et al., 2021; Sulkowski & Ignatowski, 2020). In the Church of England there was no access to churches during the first lockdown from March to July 2020 (Anon, 2020) and access was restricted to those churches that opted to provide socially distanced worship during the third lockdown from January to July 2021 (Sherwood, 2021). Surveys suggested the pandemic had led to decreased wellbeing for both clergy and lay people (McFerran & Graveling, 2021; Village & Francis, 2021c, 2022b, 2022c). Despite the obvious negative effects, there was also some evidence of spiritual awakening during this period, with many clergy and churchgoers reporting increases in prayerfulness, closeness to God, and thankfulness (Francis & Village, 2021; Francis et al., 2022; Graveling, 2023). This mix of reduced wellbeing but resilient spirituality has been reported from other religious groups (Counted et al., 2022; Osei-Tutu et al., 2021).

This paper examines the relationship between changes in spiritual wellbeing and changes in self-rated mental and physical health in a sample from the Church of England taken during the third national lockdown in 2021. The aim was to test whether those who reported better changes in spiritual wellbeing also reported better changes in health during a sudden crisis. A further question is how self-perceived changes in spiritual wellbeing related to self-perceived changes in psychological wellbeing, and whether or not any association of spiritual wellbeing on health could be entirely accounted for by differences in psychological wellbeing. First, it is necessary to show why we might expect spiritual wellbeing to be related to mental and/or physical health, and to explain and justify the concept of ‘spiritual wellbeing’ used within a committed Christian religious sample.

### Religion and Health

The relationships between religion and health have been widely studied since the 1970s, when the long-standing scepticism about linking the two domains began to be eroded by evidence from social science and health studies. The *Handbook of Religion and Health (HRH)* (Koenig et al., 2001) was a major summary of the state of knowledge to the end of the last century, and was updated within a decade (Koenig et al., 2012), illustrating the rapid growth of this field of research. Two substantial sections of the handbook, ‘Research on religion and mental health’ and ‘Research on religion and physical health’, highlight two of the key areas that have preoccupied researchers over the last five decades.

In a review article published as the *HRH* second edition was published, Koenig (2012) noted that around 80% of the research on religion/spirituality (R/S) and health involves studies on mental health. The links are likely to be more direct than for physical health, he argued, as R/S could both enhance positive emotions and reduce negative ones. His review mentions examples of over 454 studies that have demonstrated how R/S improved outcomes for people suffering a wide range of adversity from specific serious illnesses to natural disasters and war. Many studies have also shown that R/S is positively associated with positive emotions such as happiness and hope, and with enhanced wellbeing. Of 326 studies of wellbeing, only three reported negative relationships with R/S. The mechanisms by which these links are established are harder to prove, but alongside the well-studied area of religious coping mechanisms (Gall & Guirguis-Younger, 2013), Koenig includes public and private practices, which presumably include the rituals associated with particular religions that may both reinforce positive emotions and support cognitions that offer ways of combating adversity.

There are fewer studies demonstrating links between physical health and R/S, but most are related to health conditions that might be improved by reducing negative emotions such as depression or and anxiety. Health conditions where there have been studies showing improved outcomes or lower susceptibility to illness related to R/S include heart disease, strokes, dementia, diseases related to immune or endocrine functioning, and cancer. Alongside these are studies of self-rated physical health, where Koenig mentions that of 37 such studies rated most methodologically rigorous in the *HRH*, 21 reported significant positive correlations between R/S and self-rated physical health. The mechanisms by which R/S might influence physical health include the benefits from improved psychological health, social support from R/S communities, and the promotion of better health behaviour as a consequence of beliefs and values associated with R/S.

### Defining Religion and Spirituality Within Psychology and Healthcare

Alongside the emerging field of religion and health has been the emergence of spirituality as something that is related to, but distinct from, religion (Heelas & Woodhead, 2005; Roof, 2001). Early studies of religion and health emerged mainly from the USA, where Christianity was the dominant religion and ‘religiosity’ was measured by standard markers such as church attendance. As mainstream religion has declined, the notion of spirituality has become more important, though it is difficult to define and is not necessarily distinct from religion (Ammerman, 2013a, 2013b; Streib & Hood, 2016b). The first edition of the *HRH* conceived spirituality as the broader sphere within which established traditions operate, allowing for spirituality that was ‘unmoored’ to religion (Koenig et al., 2001, pp. 18–20). The trend to separate religion and spirituality, most noticeable among psychologists and health workers, has had the effect of narrowing the definition of religion to specific practices and creating broad and vague definitions of spirituality that can be indistinguishable in practice from measures of mental health (for reviews and critique see, for example, Clarke, 2009; Damiano et al., 2019; Hill et al., 2000; Klein et al., 2016; Koenig, 2012; Streib & Hood, 2016b). In some cases, the distinction has been blurred and the two terms treated almost synonymously, as do Koenig (2012) and Paloutzian and Park (2013), as critiqued by Stausberg (2015).

The Bielefeld cross-cultural study of spirituality (Streib & Hood, 2016a), which drew on samples from the US and Germany, paid particular attention to how religion and spirituality could be conceptualised and measured within a psychological framework. The authors question the use of spirituality as a construct that is separate from, or a substitute for, religion but instead suggest that spirituality is a form of religion that is both private and focused on experience. They argue that it is best used as an ‘emic’ term that individuals may use as a way of self-description or religious identity (Streib & Hood, 2016b). When individuals refer to themselves as ‘spiritual’ or to their ‘spirituality’ they may be pointing to different ideas depending on their particular context and background, but the term is nonetheless meaningful and important to them. Spirituality among those moored to Christian churches is likely to be understood partly in relation to the specific practices, such prayer and Bible reading, and partly to individual relationship with God. This notion of spirituality allows it to be defined as practices within a specific religious framework and as a self-perceived state, that is, an ‘emic’ term as suggested by Streib and Hood (2016a).

### Measuring Change in Spiritual Wellbeing, Psychological Wellbeing, and Health During the Pandemic

The outbreak of COVID-19 prompted a number of studies into the extent to which religious or spiritual factors may have helped people to cope with the pandemic. Using meta-analyses and meta-regressions, Pankowski and Wytrychiewicz-Pankowska (2023a, b) examined the relationships between various measures of religious coping and positive or negative mental health outcomes. For positive mental health measures, the results from 59 studies suggested there was a relationship between religious coping and positive mental health. For negative mental health measure, the results from 33 studies were inconclusive and suggested religious coping had little or no effect. In both papers, the authors pointed out the difficulties of combining results from different studies where the population profiles varied and were not always described adequately.

The COVID-19 pandemic produced sudden and unprecedented changes in the religious life of many churches, including the Church of England. The removal of in-church services in the first lockdown led to declines in psychological wellbeing among some clergy. The *Living Ministry* project in the Church of England has run panel surveys of clergy ordained since 2006 (Church of England, 2021). The surveys have included the Warwick Edinburgh Mental Wellbeing Scale (WEMWBS) as a measure of mental wellbeing (Tennant et al., 2007). The Panel 3 survey ran in 2021 and results were compared with the Panel 2 survey of 2019 for 340 clergy who completed both surveys. Of these, 42% reported their mental wellbeing to be worse, and average WEMWBS scores declined from 50.0 to 47.5 (McFerran & Graveling, 2021). A qualitative study based on interviews with 63 clergy in the same study supported the quantitative data and highlighted some of the reasons why pre-existing challenges to wellbeing were exacerbated during the pandemic (Graveling, 2023).

This sort of information is available from relatively few clergy. To measure the effects of the pandemic during lockdowns in a wider section of Church of England clergy and laity it was necessary to use cross-sectional surveys with items that asked about self-perceived changes in wellbeing and health since the pandemic began. This strategy was used was used in relation to psychological wellbeing in a survey during the first lockdown to produce the Index of Balanced Affect Change (TIBACh), which proved a useful tool in measuring change in positive and negative affect since the pandemic began. (Village & Francis, 2021b, 2021c). The same instrument was used in the third lockdown, in 2021, along with items asking about self-perceived changes in spiritual wellbeing and health since the pandemic began.

In order to assess changes in spiritual wellbeing within a specifically Christian context we have conceptualised the construct in terms of change in the frequency of key spiritual practices (prayer and Bible reading), in terms of relationship with the deity (trust in God), and in terms of changes in the quality of spiritual life and spiritual health. These last two items were not defined in order to allow participants to use emic definitions that would be most meaningful to themselves. To assess changes in mental and physical health we have used single general items that allow individuals to respond according to whatever aspects of health were most pertinent to themselves.

### Research Questions

1. During the third COVID-19 lockdown in England, were self-perceived changes in mental or physical health better among those who reported better changes in spiritual wellbeing?
2. Did changes in spiritual wellbeing retain predictive power for changes in self-perceived mental or physical health after controlling for changes in psychological wellbeing, as measured by The Index of Balanced Affect Change?

## Method

### Procedure

During the third lockdown in England an online survey, named *Covid-19 and Church-21,* was delivered through the Qualtrics XM platform from 22 January to 23 July 2021. It was promoted through the online and paper versions of the *Church Times*, the main newspaper of the Church of England, and directly through a number of dioceses. It was also promoted in other Anglican churches in the UK and North America, as well as is a number of other denominations. In all 5853 responded to the survey. For this study we restricted analysis to a subset of 2292 Anglicans living in England. This subset was deleted listwise by removing responses that had missing values in any of the variables used in the analyses in this paper. This left a sample of 1878, (82% of the subset). The demographics of the original subset and final samples (Table 1) were compared using contingency-table analyses and there were no statistically significant differences in any categories.

**Table 1:** Demographic characteristics of the original and final samples of Church of England respondents

	*N* =	2292^a^	1878^b^
		%	%
Sex	Male	44.9	44.7
	Female	55.1	55.3
Age	20 s	1.9	1.3
	30 s	4.6	4.2
	40 s	9.5	9.7
	50 s	20.1	20.0
	60 s	32.8	34.6
	70 s	25.8	25.7
	80 s+	5.3	4.6
Status	Lives alone	21.8	22.1
	Had virus	5.4	5.7
	Shielded	12.8	12.6
Church Tradition	Anglo-Catholic	26.7	28.8
	Broad church	54.6	51.2
	Evangelical	18.6	20.1
Charismatic	No	85.4	85.9
	Yes	14.6	14.1
Ordained	No	65.5	62.7
	Yes	34.5	37.3

^a^All Church of England respondents, ^b^ Final sample without missing values

### Sample Profiles

The final sample comprised 55% women and 45% men, the majority (55%) were in their 50 s or 60 s, and 37% were ordained (Table 1). Of the clergy, 52% were in stipendiary parochial ministry; of the laity, 22% were in some sort of authorised lay ministry. Just over a fifth (22%) reported that they lived alone, 6% reported they had definitely had the virus, and 13% that they had had to shield (that is, isolate from all social contact) for some time during the pandemic. In terms of church tradition, just over half were identified as Broad church (51%), 29% as Anglo-Catholic, and 70% as Evangelical; 14% were identified as Charismatic.

### Instruments

*Self-perceived health change* during the pandemic was assessed by two single-item scales, measuring self-perceived changes in physical and mental health during the pandemic. They were introduced by the statement: ‘Overall, how has the pandemic affected you?’ The items had a five-point response scale labelled 1 to 5 and anchored at one end ‘poorer [physical/mental] health’ and at the other ‘better [physical/mental] health’.

*Spiritual wellbeing change* during the pandemic was assessed by a five-item scale (Table 2). Four items (frequency of prayer, frequency of bible reading, trust in God, and quality of spiritual life) were in the same section of the questionnaire introduced by the rubric: ‘This section asks about how you think your various aspects of your faith, outlook, and life may have changed during the pandemic’. Each item had a five-point response scale: ‘deceased a lot’, ‘decreased a little’, ‘same’, ‘increased a little’, and ‘increased a lot’. The fifth item ‘spiritual health’ was in a different section of the questionnaire introduced by the question ‘Overall, how has the pandemic affected you?’ It had a five-point response scale labelled 1 to 5 and anchored at one end ‘poorer spiritual health’ and at the other ‘better spiritual health’. Principal Component Analysis showed the five items aligned on a single component accounting for 60% of the overall variance. Cronbach’s alpha in the final sample used in the analyses was .83, suggesting a good internal reliability (DeVellis, 2003).

**Table 2:** Items in the spiritual wellbeing change scale

	CITC	% Decreased	% Same	% Increased
Personal prayer	.68	17	35	48
Bible reading	.63	16	55	29
Quality of spiritual life	.75	27	35	38
Trust in God	.46	6	52	42
Spiritual health	.64	22	41	37

*N* = 1878

*CICT* Corrected Item Total Correlation

*Psychological wellbeing change* during the pandemic was measured using the two components of The Index of Balanced Affect Change, TIBACh, (Francis & Village, 2021; Village & Francis, 2021b, 2022c). This instrument consists of two five-item scales: Positive Affect, PA, (Happiness, Excitement, Thankfulness, Hopefulness, and Confidence) and Negative Affect, NA, (Exhaustion, Anxiety, Stress, Fatigue, and Frustration). In this sample, the scales had good internal reliability as measured by Cronbach’s alpha (PA = .77, NA = .81).

### Control Variables

*Personal variables* were sex (male = 0, female = 1) and age (by decade 18–29 = 2, 30 = 3 etc. to 80+  = 8).

*Psychological variables* were assessed using the revised version of the Francis Psychological Type and Emotional Temperament Scales (FPTETS-R) (Village & Francis, 2022a, 2023)*.* This is a 50-item instrument comprises four sets of ten forced-choice items related to each of the four components of psychological type: orientation (extraversion or introversion), perceiving process (sensing or intuition), judging process (thinking or feeling), and attitude towards the outer world (judging or perceiving), and ten items related to emotional temperament (calm or volatile). Previous studies have demonstrated that the instrument functions well in a range of church-related contexts (Village & Francis, 2023). In this sample, the alpha reliabilities were .84 for the EI scale, .79 for the SN scale, .76 for the TF scale, .82 for the JP scale, and .84 for the CV scale. Scores (rather than binary preferences) were used to indicate inclinations for extraversion, intuition, feeling, judging, and emotional volatility.

*Contextual variables* were household status (0 = living with others, 1 = living alone), virus experience (0 = not known to have had COVID-19, 1 = definitely had COVID-19), shielding history (0 = not had to shield, 1 = shielded for some of the time during the pandemic), and ordination status (0 = laity, 1 = clergy). The term shielding in the UK pandemic was used of those people who were isolated in or within homes to avoid any close contact with others because they were deemed to be especially vulnerable to the effects of COVID-19.

*Ecclesial variables* were church tradition and Charismaticism. Church tradition was assessed using a seven-point bipolar scale labelled ‘Anglo-Catholic’ at one end and ‘Evangelical’ at the other. It is a good indication of differences in belief and practice in the Church of England (Randall, 2005; Village, 2012). In the Church of England, Anglo-Catholics tend to be liturgical traditionalists but more liberal on moral issues, while the reverse is true for Evangelicals (Village, 2012, 2018b). A second similar 7-point scale was used to assess Charismaticism, a faith expression that is found across the other three traditions in the Church of England, but which is mainly associated with Evangelicals.

### Analysis

The first step of the analysis was to examine bivariate correlations between the independent variables using a correlation matrix. The second step involved hierarchical linear regression, separately on change in physical health and on change in mental health, with control and predictor variables entered in five blocks: block one, personal variables (sex and age); block two, psychological control variables (psychological type and emotional temperament); block three combined contextual control variables (virus experience, shielding experience, ordained status) and ecclesial control variables (church tradition and Charismaticism); block four, change in spiritual wellbeing; and block five, change in psychological wellbeing (positive and negative affect). The aim was to look for evidence that changes in spiritual wellbeing had a direct effect on physical or mental health changes, after controlling for other variables and allowing for changes in psychological wellbeing. In the final step, possible mediation relationships between spiritual wellbeing change and health changes via positive and negative affect were tested using the Hays Process macro (Hayes, 2013) implemented in SPSS 28 (IBM Corporation, 2021). Age, shielding, and emotional volatility were added as covariates, the number of bootstrap samples was set to 5000, and output set to 99% confidence limits.

## Results

### Bivariate Correlations

Change in spiritual wellbeing was positively correlated with change in positive affect and negatively correlated with change in negative affect and with emotional volatility (Table 3). The best psychological-type predictor of positive change in spiritual wellbeing was higher feeling scores, followed by higher intuition scores and higher extraversion scores. There was no correlation with judging scores. Change in spiritual wellbeing was also negatively correlated with emotional volatility. Change in spiritual wellbeing was higher among women than among men, among older than among younger people, among those who were living alone than among those with others in their household, and among Evangelicals and Charismatics than among those from other church traditions. Change in psychological wellbeing was correlated with psychological type and with emotional volatility in ways previously demonstrated (Village & Francis, 2021c): positive affect was negatively correlated with negative affect, emotional volatility, and judging and positively correlated with feeling, intuition, and extraversion. Some of the psychological control variables were correlated with other control variables: for example, women tended to score higher on feeling and lower on intuition than did men. For this reason, it was necessary to use multiple regression to isolate the independent effects of spiritual wellbeing change on health changes.

**Table 3:** Correlation matrix of independent variables

		1	2	3	4	5	6	7	8	9	10	11	12	13	14	15	16
1	Spiritual WB																
2	Female	.05*															
3	Age	.09***	− .02														
4	Shielded	.06**	− .01	.13^****^													
5	Lives alone	.06**	.15***	.10***	.01												
6	Had virus	.01	− .02	− .11***	− .03	− .04											
7	Ordained	− .01	− .17***	− .14***	− .06**	− .04	.03										
8	Anglo-Cath	− .04	− .08**	.03	.01	.08***	− .01	.05*									
9	Evangelical	.05*	− .07**	− .08**	− .01	− .10***	.03	.03	−.32***								
10	Charismatic	.11***	− .01	− .09***	.00	− .04	.05*	.14***	−.18***	.39***							
11	Emotionality	− .12***	.14***	− .18***	.06*	.01	.03	− .08**	.04	−.05*	−.02						
12	Extraversion	.05*	.02	.00	− .02	− .10***	− .01	.06**	− .11***	.09***	.08***	− .14***					
13	Intuition	.11***	− .08***	− .10***	.01	− .05*	.04	.24***	.01	−.01	.13***	−.06*	.18***				
14	Feeling	.13***	.19***	.01	− .02	.02	.01	.11***	− .02	− .04	.04	− .05*	.18***	.12***			
15	Judging	− .03	.00	− .01	.01	− .01	− .02	− .11***	.05	.01	− .10***	.07**	− .21***	− .43***	− .28***		
16	Neg affect	− .22***	.03	− .27***	− .01	− .04	.04	.09***	.04	− .05*	.03	.36***	− .03	.01	.01	.01	
17	Pos affect	.42***	− .05*	.11***	.02	.03	− .02	.04	− .05*	.09***	.08**	− .29***	.10***	.10***	.06*	− .06*	− .53***

*N* = 1878. *p < .05; **p < .01; ***p < .001. For an explanation of ‘shielding’ in the UK context, see text under contextual variables

### Hierarchical Linear Regression: Mental Health Change

Participants tended to report more negative mental health change during the pandemic if they were male, younger, preferred sensing over intuition, were more emotionally volatile, and lived alone (Table 4, model 3). Spiritual wellbeing change was positively correlated with better mental health changes after controlling for personal, psychological, ecclesial, and contextual variables (Table 4, model 4). Predisposition to emotional volatility was strongly negatively correlated with better mental health change, but the effect was reduced when adding changes in positive or negative affect, as was the effect of spiritual wellbeing (Table 4, model 5). Increase in negative affect predicted decrease in mental health and increase in positive affect predicted positive change in mental health. This suggests the effect of change in spiritual wellbeing on mental health might have been partially mediated via its effect on change in psychological wellbeing. Mediation analysis confirmed this (Table 6): after controlling for age, shielding and emotional volatility, the indirect effects of change in spiritual wellbeing on mental health change through change in positive and negative affect accounted for 70% of the total effect.

**Table 4:** Hierarchical linear regression of mental health change scores

	1	2	3	4	5
Female	.01	.07**	.06*	.05*	.06**
Age	.24***	.18***	.18***	.16***	.10*
Extraversion		− .01	.00	.00	.00
Sensing		− .05*	− .06*	− .03	− .02
Thinking		.03	.02	.05	.03
Judging		.02	.02	.01	.01
Emotional volatility		− .35***	− .35***	− .33***	− .19***
Shielded			− .03	− .05*	− .05**
Lives alone			.05*	.04	.03
Had COVID-19			− .02	− .02	− .02
Ordained			− .04	− .03	.00
Charismatic			.01	− .01	− .02
Evangelical			.00	− .01	− .03
Spiritual wellbeing				.22***	.07***
Negative affect					− .32***
Positive affect					.26***

*N* = 1878. *p < .05; **p < .01; ***p < .001. For an explanation of ‘shielding’ in the UK context, see text under contextual variables

### Hierarchical Linear Regression: Physical Health Change

Participants tended to report more positive mental health change during the pandemic if they preferred extraversion to introversion, judging to perceiving, or were evangelical. More negative physical health change was associated with emotional volatility, having had to shield, or if they had had the virus, but not if they lived alone (Table 5, model 3). The age effect decreased when emotional volatility was added to the model, in-line with the negative correlation between age and emotional volatility identified in Table 3. Evangelicals seemed to report better physical health changes than others, though this may have been because they also reported better changes in psychological wellbeing. Spiritual wellbeing change was positively associated with better physical health changes (Table 5, model 4), an effect that persisted after adding psychological wellbeing changes (Table 5, model 5). Extraversion and judging both had small but statistically significant positive effects on physical health changes which persisted after psychological wellbeing and spiritual wellbeing were included in the model. Predisposition to emotional volatility was strongly negatively correlated with better physical health change, but the effect was reduced when adding changes in negative affect, as was the effect of spiritual wellbeing (Table 5, model 5). Increase in negative affect predicted decrease in physical health; increase in positive affect had small positive effect. This suggests the effects of spiritual wellbeing and emotional volatility on physical health might have been partially mediated by their effects on negative affect. Mediation analysis confirmed this (Table 6): after controlling for age, shielding and emotional volatility, the indirect effects of change in spiritual wellbeing on mental health change through positive and negative affect accounted for 40% of the total effect, and only the indirect effect of negative affect was statistically significant.

**Table 5:** Hierarchical linear regression of physical health change scores

	1	2	3	4	5
Female	− .01	.02	.01	.00	.00
Age	.06**	.03	.03	.01	− .02
Extraversion		.07**	.07**	.07**	.07**
Sensing		.00	− .02	.00	.01
Thinking		− .01	− .02	.00	.00
Judging		.07**	.07**	.06*	.07**
Emotional volatility		− .19***	− .18***	− .17***	− .09***
Shielded			− .07**	− .08**	− .08**
Lives alone			.02	.01	.01
Had COVID-19			− .06**	− .06**	− .06**
Ordained			− .04	− .03	− .01
Charismatic			− .04	− .05	− .05
Evangelical			.06*	.06*	.04
Spiritual wellbeing				.16***	.10***
Negative affect					− .22***
Positive affect					.07**

*N* = 1878. *p < .05; **p < .01; ***p < .001. For an explanation of ‘shielding’ in the UK text, see text under contextual variables

**Table 6:** Mediation analyses for effects of spiritual wellbeing on health changes via psychological wellbeing variables

	Mental health change	Physical health change
Total effect	.052 (.005)**	.039 (.006)**
Direct effect	.016 (.005)**	.023 (.006)**
Indirect effect	.036 (.003)**	.016 (.003)**
Indirect: via negative affect	.014 (.002)**	.010 (.002)**
Indirect via positive affect	.021 (.003)**	.006 (.003)

Figures in parentheses are SE. **p < .01

## Discussion and Conclusions

This study examined self-reported changes in spiritual wellbeing, psychological wellbeing, physical health, and mental health among a sample of 1878 members of the Church of England during the third national COVID-19 lockdown in 2021. The results provided answers to the two research questions.

The first research question asked whether self-perceived changes in mental or physical health were better among those who reported better changes in spiritual wellbeing. The data demonstrated that better change in spiritual wellbeing was positively correlated with both better change in mental health and better change in physical health during the pandemic lockdown. This finding is in-line with the many studies that have demonstrated, or argued for, the positive links between religion and or spirituality and a range of aspects of health (Koenig, 2012; Koenig et al., 2012), including during the COVID-19 pandemic (Hart & Koenig, 2020; Koenig, 2020). The direction of effect, and any causal links, are difficult to establish in this sort of cross-sectional study, but if causal links existed it might have been because this religiously committed sample was drawing on spiritual coping mechanisms that were linked to their Christian faith. Unlike studies of the general population in normal times, this was a sample where religion was likely to have been generally salient for all respondents, though the shared religious life associated with gathering for worship had been radically altered and curtailed (Bryson et al., 2020; Edelman et al., 2021). In these circumstances it may have been the specifically private practices (such as prayer or reading Scripture) and trust in God that may have been the most important aspects of faith that were likely to prove helpful for promoting mental health.

The positive association between change in spiritual wellbeing and change in physical health is more difficult to explain, especially as the nature of physical deterioration or improvement was not specified. The survey did ask about aspects linked to physical health such as exercise and eating behaviours, and while many reported no change, some reported both increases and decreases in fitness and consumption (Village & Francis, 2021a). The isolation of lockdown was both an opportunity to develop new helpful patterns of behaviour that might benefit health, but also a threat to losing regular healthy pre-pandemic habits. Spiritual life may have enabled some to cope with the changes of lockdowns in ways that promoted more disciplined, healthy behaviours.

The second research question asked whether changes in spiritual wellbeing retained predictive power for changes in self-perceived mental or physical health after controlling for changes in psychological wellbeing. The data demonstrated that change in psychological wellbeing as measured here was strongly related to self-reported changes in mental health, so it might have been expected that any effect of spiritual wellbeing on mental health would disappear after controlling for psychological wellbeing. The effect was certainly reduced, but not eliminated, suggesting that spiritual wellbeing may have been important in promoting mental health during the pandemic. This is an important finding in relation to studies that have argued that spiritual wellbeing has tended to be elided with psychological wellbeing in some conceptualisations. Here we show that specifically spiritual notions of wellbeing may be important in maintaining overall mental health (at least among religiously engaged participants). The effect was largely indirect: after controlling for age, shielding and emotional volatility, the indirect effects of change in spiritual wellbeing on mental health change through positive and negative affect accounted for 70% of the total effect. Spiritual wellbeing may promote positive affect and reduce negative affect, leading to improved overall mental health outcomes during a traumatic time such as a pandemic.

With physical health, there was some indirect effect of change in spiritual wellbeing via reduction in negative affect, but the main effect was direct rather than mediated. Given that the physical health measure was self-reported, it might be that spiritual wellbeing was offering the means of helping individuals to accept or cope better with deteriorating physical health during the pandemic.

### Limitations of the Study

This was a convenience sample of Anglicans living in England rather than a random probability sample, so the findings may not apply to the Church of England as a whole. Membership of the Church of England is not concisely defined, so the demographic characterises of the whole population are not known for certain. Where similar convenience samples collected in the same way as in this study have been compared to known subgroups (e.g. clergy) the results suggest the convenience samples are reasonably representative (Village, 2018a).

This survey attempted to measure changes in spiritual wellbeing, psychological wellbeing, physical health, and mental health after the onset of an unexpected pandemic and the subsequent lockdowns. As such it relied on cross-sectional rather than longitudinal measures of wellbeing and health. These were self-reported measures, and therefore not as robust as would have been the case if we were able to measure these variables longitudinally before and after the onset of the pandemic. Self-report measures are useful, but not as accurate or rigorous as other ways of measuring mental and physical health that use larger batteries of items that would not have been possible in a survey that also measured a wide range of aspect of faith, attitudes, and worship practice. Future studies in times of crisis in the Church of England would be helped by having on-going long-term panel studies similar to that used on clergy but applied to lay people as well.
